# Global scientific research commons under the Nagoya Protocol: Towards a collaborative economy model for the sharing of basic research assets

**DOI:** 10.1016/j.envsci.2015.08.006

**Published:** 2016-01

**Authors:** Tom Dedeurwaerdere, Paolo Melindi-Ghidi, Arianna Broggiato

**Affiliations:** a Université catholique de Louvain, Belgium; b GREQAM Aix-Marseille Université, France

**Keywords:** Collaborative economy, Open access policies, Access and benefit sharing, Biodiversity research, Culture collections

## Abstract

This paper aims to get a better understanding of the motivational and transaction cost features of building global scientific research commons, with a view to contributing to the debate on the design of appropriate policy measures under the recently adopted Nagoya Protocol. For this purpose, the paper analyses the results of a world-wide survey of managers and users of microbial culture collections, which focused on the role of social and internalized motivations, organizational networks and external incentives in promoting the public availability of upstream research assets. Overall, the study confirms the hypotheses of the social production model of information and shareable goods, but it also shows the need to complete this model. For the sharing of materials, the underlying collaborative economy in excess capacity plays a key role in addition to the social production, while for data, competitive pressures amongst scientists tend to play a bigger role.

## Highlights

• Develops a collaborative economy model for sharing research materials for biodiversity research.
• Presents a world-wide survey on collaborative practices in global research for sustainability.
• Shows the limits of relying on internalized motivations only to build a collaborative economy.
• Provides inputs for the implementation of the Nagoya Protocol for non-commercial research.

## 1 Building global scientific research commons for biodiversity research

The importance of international cooperation for basic biodiversity research has been recognized since the very first debates on the design of global environmental governance regimes. Principle 20 of the 1972 Stockholm Declaration of the United Nations Conference on the Human Environment underlines that the “free flow of up-to-date scientific information and transfer of experience must be supported and assisted, to facilitate the solution of environmental problems” (UN Declaration on the Human Environment, 1972). However, in spite of important international initiatives, such as the Global Biodiversity Information Facility (GBIF) or the International Nucleotide Sequence Database Collaboration (INSDC), no clear legal and institutional framework has emerged to support such cooperation. Moreover, an increase in restrictions on global access to basic research assets has been documented in specific fields of life science research, with a potential detrimental impact on access to scientific publications, research samples and genomic databases (Jinnah and Jungcurt, 2009: 464; Reichman and Okediji, 2012). In addition, competitive pressures amongst scientists tend to counter-balance the social norms of scientific research communities, leading for example to delays in release of scientific results and research data (Dasgupta and David, 1994).

In this context, the Nagoya Protocol (Nagoya Protocol on Access to Genetic Resources and the Fair and Equitable Sharing of Benefits Arising from Their Utilization to the Convention on Biological Diversity, 2010) offers an important opportunity for contributing to the emergence of an international institutional framework for biodiversity research. On the one hand, the implementation of the Protocol might add to the existing challenges for the functioning of the commons, but on the other, the Protocol also presents opportunities for a mutually supportive implementation between the existing practices of sharing of essential research assets and the access and benefit sharing regime. Indeed, as can be seen in particular in the annex to the Protocol, a broad variety of non-monetary benefit-sharing measures are envisioned as means to organize a fair and equitable sharing of research benefits between participating countries, which can be used to create a collaborative framework for the upstream dimensions of the research cycle. Moreover, different articles of the Protocol, such as articles 8, 10 and 11 explicitly address the issue of non-commercial and/or transboundary research cooperation.

Governments and research institutions throughout the world have already taken steps in the direction of such a mutually supportive implementation. Examples are the legislations on facilitated access to biological resources for non-commercial research in Australia or Brazil, amongst others, and the recommendations for accessing biological resources in basic research adopted by the German Research Foundation, DFG. However, these steps deal with the regulation of case by case bilateral transactions over single research assets and not with the building of global scientific infrastructures. In general, with the notable exception of the International Treaty on Plant Genetic Resources, little attention has been given to the likely consequences of the implementation of the Protocol for such global collaborations.

With a view to contributing to a better understanding of this issue, this paper analyses the functioning of global collaborations for biodiversity research in the specific field of microbiology. The field of microbiology has a long history of global collaboration, especially between the ex situ collections of microbial organisms that are member of the World Federation for Culture Collections (WFCC, cf. www.wfcc.info). The World Federation is a multidisciplinary commission of the International Union of Biological Sciences (IUBS) and has been actively promoting collaboration amongst the major collections in the world, in particular through the establishment of common standards for exchange and the building of an information network between the collections and their users (the so-called World Data Centre for Microorganisms). Therefore the case of the microbial culture collections is particularly interesting, as these collections have a well-established history of managing global scientific research commons. Within this specific context, this study aims to analyze what factors contribute to the public availability of the upstream research assets managed by the culture collections, including upstream research materials and associated genomic data.

The paper is organized as follows. First, some major challenges for organizing global collaboration with microbial resources are presented (Section 2). Second, some of the limits of the conventional public economy approach for understanding global commons are analyzed and the principles of an alternative, collaborative economy model, outlined (Section 3). Third, the paper analyses the functioning of this model through a survey of two existing practices of building global commons: sharing of basic research materials between public culture collections and public deposits of genomic information by the collection managers and/or scientists contributing materials to the collections (Section 4). The paper concludes with an overview of some options and best practices that result from the analysis. These research results show the need to consider a broad interpretation of the notion of non-commercial use in the implementation of the Protocol, in order to preserve these commons based exchange practices that are essential to global cooperation for basic biodiversity research (Section 5).

## 2 Global scientific research commons in microbial resources and associated genomic data

### 2.1 Use of microbial diversity for public health, food security and biodiversity conservation

Microorganisms are supporting the health of most ecosystems on earth and play a key role in many important issues, such as agriculture and food production and human health. For instance, microorganisms play a major role in soil fertility and are employed in disease diagnostics, efficacy testing of drugs, and vaccine production amongst others. Furthermore, microorganisms play a direct role in widely used biotechnology applications, which include the biological control of pests and diseases in agriculture and horticulture, production of natural products for pharmaceutical, food and other applications, bioremediation and detoxification of wastes.

Both private and public sector organizations collect, use and distribute microorganisms on a massive scale. The global market value for microbial products – used as biopesticides in agriculture as well as in chemical production – is an estimated $156 billion in 2011 with an expected increase to more than $259 billion in 2016 (BCC, 2011). Nevertheless, the overall market value is likely to be much higher, as the direct selling of microorganisms only represents a tiny part of the overall value of microorganisms as crucial intermediaries in basic and applied research. In addition, on average, over half a million cultured microbial organisms are distributed through various public service culture collections which conserve and distribute microbial organisms for basic and applied research purposes. Although the major part is distributed to public sector organizations (77% on average), a substantial part is also provided to for-profit private sector organizations (23% on average) (Dedeurwaerdere et al., 2012a).

Public service culture collections link academia, industry, government and international knowledge providers and users of microbial material. Although the utilization of the materials held in the collection is subject to the access and benefit sharing obligations of the country of origin of the isolates, the question of the full bundle of legal ownership rights over these is highly context specific. Nevertheless, most public collections do not claim any downstream ownership rights on the materials, which they keep in “custody” or “in trust” for the entire humankind. In practice, the materials are distributed against the payment of a fee in order to cover, in part, the additional administrative costs generated by the management of the distribution system.

The role and functions of the microbial collections as a basic life science research infrastructure bears a lot of similarities with other ex situ collections, especially in the field of animal and plant genetic resources, which have been studied elsewhere (Fowler et al., 2001, Gollin et al., 2000). However, two important features are specific to the microbial collections. First, microbial organisms have extremely high mutation rates upon reproduction (Dijkshoorn et al., 2010). As a result, there is no equivalent to the relatively well-defined species concept for plants and animals. Therefore, microbial science, and private sector research and development, depend to a large extent on the purified cultured organisms held in the microbial ex situ collections. Second, without globally accepted standards and quality control of microbial holdings, entire families of clones of collected microorganisms can be contaminated, as happened in the 1960s with the contamination of microbial cell lines for cancer research, which has led to over a decade of invalid scientific publications (Stern, 2004).

However, the vast majority of microbial diversity is yet to be discovered (estimated 90–95%). In addition, the combination of the high cost of conservation of purified microbes and the very high intra-species diversity makes it impossible for one collection to cover the entire breadth of microbial diversity, even for a specific set of microbial species. Intense collaboration and exchange amongst public culture collections is therefore a necessary consequence of this situation. In the more recent history, these global collaborations between the culture collections have been expanded to the public databases containing information on the country of origin, scientific publications related to the microbial holdings of the collections and automatic linkage to associated genomic information available through open access databases (Dawyndt et al., 2006, Reichman et al., 2015).

### 2.2 Challenges regarding the public availability of upstream research assets

The globally distributed infrastructure of culture collections has led to major scientific progress and technological innovations in the past, covering food security, environmental management, biotechnology and health amongst others. However, due to pressures on public funding (Stackebrandt, 2011) and an increasing overlap of basic and applied commercial research (Cook-Deegan and Dedeurwaerdere, 2006), the public availability of both microbial research materials and the associated data has increasingly come under pressure. Three major challenges illustrate this increasing pressure.

The first challenge is related to the deposit and availability of microorganisms for further downstream research. Currently, over 200,000 new samples of microorganisms are deposited each year in the collections of the World Federation for Culture Collections, which are collected from natural environments in all geographical regions of the world (Dedeurwaerdere et al., 2012a). However, this only represents a tiny fraction of new microorganisms that are yearly discovered and referred to in published research. A 2008 survey preformed on 8 European Microbiological Journals aptly illustrates this first challenge (Stackebrandt, 2010). In the 835 articles reporting research on a total of 20,200 new isolates, only less than 1% of these isolates were deposited in public culture collections. In addition, an important portion of these strains already died or was included in a patent application process and therefore became unavailable. Even though it seems reasonable to operate a selection amongst these isolates, the majority of paper authors did not deposit any materials, which is very worrisome as conservation and availability of a selection of these materials is necessary to replicate the research findings and for follow-on research (Stackebrandt, 2010). However, except for some journals such as Nature, the recommendation given by most scholarly journals to deposit research materials in well recognized repositories is not strictly implemented and thereby left to the discretion of the researchers. Even though major culture collection networks have offered to accept all materials during or shortly after publication, there is no clear reversal of this trend, in particular due to the increasing competitive pressures on the scientists.

A second challenge is related to the availability of the research data, in particular the environmental and genetic sequence data associated with the microbial strains. As noted by many scholars, maximizing open access to basic data sets is essential for the rapid translation of research results into knowledge, products and procedures to improve matters of general interest (Stiglitz et al., 2000, Dawyndt et al., 2006). In response, various high-level policy initiatives have supported open availability of research results, such as the compulsory open access policies of the US National Institutes of Health and the European Research Council, or the Bermuda principles for rapid and public release of DNA sequence data, which have been endorsed by major science funders and publishers. However, in the same time, scientists often make data available only after major delays, even after the publication of the first scientific results from this data. The latter has been well documented for the case of access to research samples and genomic data in the life sciences (Dedeurwaerdere, 2006, Walsh et al., 2007). As shown by a major cross-disciplinary survey, the main reasons for lack of sharing are limited time and funding for infrastructure support (Tenopir et al., 2011).

The adoption of the Nagoya Protocol might have an impact on both these challenges and add a third layer of barriers to the public availability of upstream research assets. In principle, the implementation of the Protocol can be supportive of the existing practices of global cooperation in biodiversity research. The latter is in line with one of the objectives of the Protocol, which is to foster biodiversity research, as stated in several of its provisions (Dedeurwaerdere, 2010b). However, the implementation of the Protocol also requires a clear tracking and monitoring of the access agreements and of the utilization of the accessed materials by all the parties involved in international exchanges, which might dramatically increase the transaction costs for the operation of the commons. Nevertheless, in the field of the exchange of materials, many of the collections that are member of the WFCC have already taken steps to contribute to such tracking and monitoring, without major impact on the hampering of the exchanges. In particular, the use of a formal Material Transfer Agreement and the documentation of countries of origin is an established practice, even though new constraints for such monitoring and/or documentation might arise in the context of the implementation of the Protocol (Dedeurwaerdere, 2010a). In contrast, the fate of public availability of environmental and genomic data, that are associated with the microbial holdings, is still highly uncertain (Greiber, 2014).

This papers aims to analyze how and to what extent the existing practices of the collections are able to overcome these three challenges to the public availability of upstream research assets. The next section addresses the first two of these challenges, based on the literature on the models for overcoming collective action failures in scientific research commons. The detailed discussion of the third challenge falls partially out of the scope of this paper. Nevertheless, two specific questions will be addressed in relation to the third challenge. First, is there evidence of any impact of existing access and benefit sharing regulation on the sharing practices; and, second, what best practice guidelines can be derived from the results of the analysis in this paper that can serve as input into the discussions on the implementation of the Protocol?

## 3 Overcoming collective action failures through social production and collaborative economy models

Economic theory of public goods provision highlights major collective action challenges for organizing collaborations in global commons. In this context, global commons are defined as global research assets governed by a group of information producers and/or users under non-exclusive use conditions (Benkler, 2006). Two core arguments show potential difficulties for the long-term sustainability of such commons. The first is based on the so-called prisoners’ dilemma, which shows that, without clear guarantees on the other players’ cooperative behaviour, agents will not cooperate spontaneously, even if greater long-term benefit could be achieved from cooperation (Ostrom, 1998). The second argument is based on the free rider problem in public good provision, which shows that, without enforcement measures, some people will attempt to benefit from public goods that are produced, without contributing, as it is publicly available once it is produced by others (Sandler, 2004). As a result, even if institutional rules are found to overcome the prisoners’ dilemma by the involvement of a core group of contributors, the overall provision of the public good can still be less than what could be the case if all the players would contribute in a fair and equitable manner.

A conventional solution to these problems is to introduce an external state authority that imposes general interest and long-term objectives on the individuals (Hardin, 1968). For the organization of global research commons this would imply to create a global authority, through a multilateral agreement, with jurisdiction over the scientific research assets and which would act as an external rule enforcer. Important examples of such a solution are the International Treaty on Plant Genetic Resources for Food and Agriculture, and the Global Influenza Surveillance and Response System of the WHO. Whenever such a global state authority is not available, an alternative solution proposed in the literature is to revert to private appropriation of the research assets under exclusive access regimes (Hardin, 1968) and organize collaboration on market-based principles. In such a market-based model, global research infrastructures can be formed spontaneously based on voluntary initiatives using competitive monetary pricing based on willingness to pay. An example of the latter are specialized service collections selling clones of microbial materials that can be used by industry or universities for specific research purposes, such as Forintek Inc., which is a global culture collection that provides research services and materials to the timber industry on a for-profit basis (Stromberg et al., 2013).

The global state-like and the global market-like solution for organizing collaborative research should however not be regarded as the only possible institutional models. In particular, these two solutions do not seem to reflect adequately the research collaborations amongst culture collections reviewed above, which are sustainable even in the absence of exclusive market-like access regimes or the absence of a global external state-like authority. For example, many essential knowledge assets for scientific research in microbiology are made available by distributed networks of collections that share these resources on a partially or totally non-exclusive basis. The latter points to the relevance of a third solution, which is based on the operation of social networks and the internalization of collective goals in so-called social production models of knowledge (Benkler, 2006). Scholars of knowledge commons have analyzed this social production model in more detail for the specific cases of the social production of information on the one hand and the exchange of shareable goods on the other. These two cases will be highlighted in the next two sub-sections.

### 3.1 Social production model for informational knowledge commons

Systematic research on generic design principles of governance of knowledge commons with informational assets, such as genomic data, software or scholarly publications, has identified a set of design principles for successful governance arrangements. First, this research has shown that, in such commons, participants are driven more by social motivations (especially reputational and social identity related motivations) and internalized motivations (such as the science ethos or other collective values that have been internalized and endorsed by the actors) (Ryan and Deci, 2000), than by the prospect of direct monetary rewards (Schweik and English, 2013, Dedeurwaerdere, 2012, David and Shapiro, 2008). As a result, global scientific research commons are in practice governed by a set of mixed incentive schemes (Benkler, 2006), which include both self-interested behavioural incentives (such as direct reciprocity or monetary rewards) and other-regarding behavioural incentives (such as the science community's publication norms and individual scientists’ personal values), along with collective rules signalling trusted knowledge providers in the informational economy (Lessig, 2008).

A second common feature is related to the transactional features of collaboration with information assets. Indeed, not all modes of organization with distributed information assets lead to cost-effective transactions (as compared to the option of a centralized state-based solution). The adoption of distributed (exploiting the power of digital networks for coordination) and fine-grained modular architectures (based on a division of labour amongst geographically distributed components, each specializing in different sub-tasks that only ask a small additional effort/cost) is a second major institutional feature bearing on the success of information-based knowledge commons in digital networks. According to the scholars of knowledge commons, what is most important for the operation of such a cost-effective transaction system, is the sharing of common norms within a network organization, in order to enable many participants to effectively pool their efforts and contributions, notwithstanding the fact that these contributions may vary in quality, focus, timing, and geographical location (Benkler, 2006).

Scholars of scientific research commons have analyzed this social production model in more detail for the specific cases of practices of information-sharing throughout the scientific research process, such as intermediary results or new yet unpublished data and bio-materials. They show that the sharing practices are highly context dependent because of the different trade-offs that exits between the communal norms of scientists and the competitive incentives for researchers during the research process. In particular, researchers might keep information secret during pre-publication stage, or even after the publication of the first research results, with a view to gaining possible future reputational benefits from further downstream research with that same information (Dasgupta and David, 1994). In the case of so-called *general sharing* of intermediary results through public open research infrastructures, which is the topic of this paper, sharing will depend on the presence of contextual factors that outweigh the competitive incentives. As shown in an empirical analysis of bio-scientists in Germany and the United Kingdom, important factors that determine the level of *general sharing* in this context are the value of feedback (such as providing complementary information on research results), beliefs about proper acknowledgement and the level of competition (Haeussler et al., 2014).

As highlighted in introduction, such competitive pressures have also increased in the case of the researchers and the managers who decide upon the sharing practices in the culture collections. Therefore, although most of the assets in the collections are situated high upstream in the research cycle, the context dependent benefits are also likely to be crucial to promote sharing practices with these basic research assets. Two of these factors are particularly strong in the case of the sharing of materials, which are, first, the increase in scientific feedback resulting from the deposit of materials (such as a clear increase in citation rates of articles when these refer to materials deposited in public collections) (Furman and Stern, 2011); and the benefits for small collections that result from participating in a distributed network of collections with proper standards of tracking and acknowledging the origin of the assets that they make available (Stern, 2004). In contrast, in the case of data, because of the difficulty to track downstream uses, such contextual benefits are virtually absent. Therefore, in the case of data, in addition to the role of the communal norms of science, external rules of collective action, such as mandatory deposit of genomic data, are expected to play a much more important role in the promotion of the sharing practices.

### 3.2 Collaborative economy model for the sharing of materials

The literature on the social production model combines insights from sociology of science and economics of science and innovation. Although many of the arguments can be transposed to the case of the sharing of basic research materials through public infrastructures, the model remains insufficient to capture some of the economic features of the sharing of the microbial materials. Indeed, differences in distribution costs between information and materials impose different economic constraints on the sharing practices.

The key difference in distribution costs between the biological and the informational resources is related to the nature of the output. In the case of the informational goods, even though the original production costs might be high, the use of digital technologies allows to operate with near zero marginal distribution and storage costs. Therefore, in the absence of intellectual property rights, the sharing of the information goods as public goods generates very few additional transaction costs. In contrast, in the case of the sharing of biological resources, the marginal storage and distribution costs can be important, as additional physical storage is needed for every additional item, and technical staff is required for managing the long-term storage and quality management requirements for publicly available microbial research materials. Therefore, additional governance mechanisms are needed to use the social production model for the sharing of material assets.

The main feature that allows keeping the transaction costs for the sharing of basic research materials low, is that most biological materials have multiple uses (Dijkshoorn et al., 2010). This multi-functionality generates a high amount of excess capacity that can be made available to other researchers. Indeed, researchers and culture collection managers, who obtain funding for storage facilities and technical staff to conduct research in a certain research field, can easily duplicate these same purified microbial organisms – through cloning – for other research demands in entirely different locations, without generating important additional costs beyond some additional administrative costs related to the ordering and shipping of the materials.

However, in contrast to the informational knowledge commons, shareable excess capacity is depletable upon use and therefore a certain selection mechanism of potential users needs to be put in place. Depending on the cost of organizing such a mechanism and the type of benefits that can be expected, researchers and culture managers can opt for a social mechanism for selecting the users (through using social networks and social production tools) or a secondary market in the excess capacity (through limiting access and use, pricing the transactions and monitoring the agreed upon uses).

The governance of material goods that are shared through the use of digital networks, on the basis of the excess capacity of a given private good, has been extensively studied over the last decade in the literature on the so-called collaborative economy (Botsman and Rogers, 2010). As shown in this literature, the choice between a social mechanism for organizing the sharing and a secondary market will depend on a set of transaction and motivational features of the exchange (Benkler, 2004). In the case of the market solution for organizing the selection of users of the excess capacity, an important motivation is the monetary retribution, but this has to be weighed against the possible transaction costs generated by the pricing of the goods, the monitoring of the uses of the excess capacity and the possible loss of social benefits. In the case of a social mechanism, the motivations are more of a social and personal nature, such as contributing to scientific research or increasing one's reputation as a good scientist. The transaction costs, on the other hand, will depend on the presence or absence of pre-existing social norms and organizational networks for monitoring the exchanges, and the possible opportunity costs of not sharing the materials, such as possible loss of a good reputation in the social networks.

In the case of the culture collections, it can therefore be expected that the international federations play an important role in the sharing of the research materials. Indeed, these federations are intensively involved in the building and enforcing of common quality standards for the sharing of materials. For instance, the World Federation requires to subscribe to minimum quality standards as a condition for membership, while the regional federations organize intense cooperation amongst the member collections for the improvement of these and other standards. Such common rules for quality management facilitate the identification of trustworthy providers and users in a cost-effective manner, which is important, seen the physical limits (both in the terms of human resources for cloning and storage capacity) on the overall amount of excess capacity that can be made available.

## 4 Data collection, empirical model and mixed method approach

Structured questionnaires with 26 closed-end questions on detailed patterns of provision and reception of materials, motivations and benefits, and governance were administered through a web-based survey tool to a representative sub-sample of 191 culture collection researchers and culture collections managers.

### 4.1 Details of the data collection

The first round of surveying was administered to the participants of the 13th International Conference on Culture Collections (ICCC 13), September 23rd to 27th 2013 in Beijing. A second round was organized between October 2013 and February 2014 with a series of additional surveys with the members of the US, the EU and the Latin American Federation of Culture Collections, with a view to having a balanced geographical representation of the sample. In total 191 responses were received (121 from the Beijing conference, amongst the approximatively 500 participants; and 70 from the email invitations sent to the 3 regional federations). As shown in Table 1, the resulting sample is representative of the geographical balance and organizational types of the overall population of culture collections that are registered on the database of the World Federation of Culture Collections (cf. http://www.wdcm.org/). Even though some public collections are still in the process of becoming member of the WFCC, the composition of the WFCC membership is nevertheless an excellent approximation of the types of collections and practices amongst the exiting public culture collections.

Prior to administering the structured questionnaire, qualitative interviews were arranged with the 2013 president of the World Federation of Culture Collections (4th June and 29th August 2013) and a meeting was organized with various culture collection representatives to discuss the goals of the survey, and make a quality check of the questions, on 22nd May 2013. Face to face interviews with 11 culture collection managers on sharing of materials were held between 23rd and 26th September 2013 at the ICCC-2013 conference. Finally a workshop was organized on 25th and 26th September 2014 to discuss the specific issues related to data sharing in relation to the Protocol (for the workshop report (Greiber, 2014)).

### 4.2 Hypotheses and empirical model

**Fig. 1:**
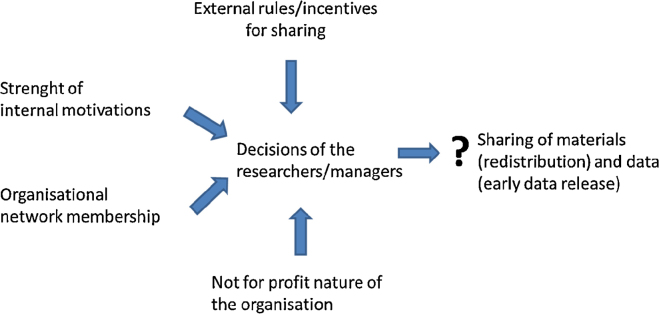
Assessing the role of internalized motivations, external rules/incentives, non-market orientation and organizational networks in global knowledge commons.

Fig. 1 schematically represents the explanatory variables that have been addressed in the survey to better understand the role of the social production and the collaborative economy model in the public availability of basic microbial research materials and associated genomic data. Each of these variables refers to different real-world features, depending on the application of the model to the sharing of research materials or to the public availability of associated genomic data. The intermediary variable “decisions of the researchers/managers” represents the core group of culture collection managers and scientists that are directly involved in handling the received samples, storage and, in an important number of cases, initial research. This core group sets the general rules in the culture collections on sharing of materials and data, taking into account the applicable legal rules and the applicable rules of their host institutions.

For materials, both variables related to the social production model and variables related to the collaborative economy model are expected to be significant. Indeed, for materials, distribution has a marginal cost to all the players and the sharing of the excess capacity through a social/collaborative mechanism requires intense coordination and trust building. Therefore, the variable “organizational network membership”, which is related to the collaborative economy model is expected to be positively correlated. The strength of internalized motivations and the not for profit nature of the organization play a role both in the social production model and the collaborative economy model and are therefore expected to be strongly correlated, while direct governmental regulation is expected to be negatively correlated.

For data, based on the social production model, the strength of internalized motivations and the not for profit nature of the organization are expected to be correlated positively with data sharing. As distribution has no marginal cost to the players, the variables related to the collaborative economy model are not expected to impact on the data sharing.

In the case of data, as reported above, due to the relative lack of direct contextual benefits from sharing of data (as compared to materials), important trade-offs between the competitive pressures and the social production model are highly likely, as some scientists expect to extract reputational rents from temporary secrecy, providing them the opportunity of being the first to publish certain results. In particular, culture collection managers face reluctance from scientists to participate to early data release associated with the microbial materials, through the culture collections data portals or through public genomic databases. Therefore, additional external rules from publishers are expected to be positively correlated with the data sharing.

Finally, the impact of access and benefit sharing regulation (ABS) is expected to depend upon the compatibility with the social production and collaborative economy model. In the case of materials, there is already a long tradition with Material Transfer Agreements for sharing, which explicitly consider access and benefit sharing. Therefore, the impact is expected to be positive or neutral. In contrast, in the case of data, there exists a high degree of legal uncertainly on the way and the possibility to reach a mutual compatibility between the public availability and the implementation of the Protocol. Therefore a negative correlation is expected.

To assess the role of these variables on the decision to allow further downstream sharing of materials and/or to deposit data on public genomic databases upon or prior to publishing, the following two regression models were developed:

• A first model on sharing of materials. This first model tests if the permission to share materials is correlated with
  ∘ internalized motivations: duty as a core motivation to provide materials (variable: Materials prov duty);
  ∘ external rules/incentives for sharing: rules on access and benefit sharing applicable to materials regulated by the country (variable: ABS country) or the organization (variable: ABS organization); the government provides incentives for sharing of materials (variable: Gov. incentives materials) and/or imposes the rules on conditions of transfer of materials (variable: Gov. rules materials);
  ∘ organizational network membership: membership of regional/international culture collection federations (variable: Networks no);
  ∘ Not for profit nature of the organization (variable: For profit)
  To account for possible restrictions imposed by the providers of the materials, 3 sub-models are developed to check the permission to share if the materials are received from 3 different categories of organizations: from other culture collections, from governmental research institutes or from university research laboratories. As similar variables are likely to play a role in these 3 sub-models, a joint estimation of the three outcome variables is appropriate. Therefore a multi-variate probit model has been used (for the justification of the choice of a probit, cf. comments below).
• A second model addresses the early release of genomic data by research and managers of the culture collections, through their deposit on external genomic databases, mainly the public databases from the INSDC (International Nucleotide Sequence Database Collaboration). This second model tests if the early release of genomic data is correlated with:
  ∘ internalized motivations: duty as a core motivation to provide materials (variable: Data prov duty)
  ∘ external rules/incentives for sharing: rules on access and benefit sharing applicable to data and information regulated by the country (variable: ABS country) or the organization (variable: ABS organization); the government imposes the rules on conditions of transfer of data (variable: Gov. rules data); publishers mandate data provision to public online databases (variable: Publishers’ rules); scientists and/or national public authorities are consulted when making decisions on transfer of data (variables Consult nat public authorities and Consult scientists); perceived importance of employers’ role in creating willingness to share (variable Perc employers role).
  ∘ Not for profit nature of the organization (variable: For profit)

With a view to verifying the consistency of the results with previous studies of the distribution patterns of the culture collections (Dedeurwaerdere et al., 2009, Stromberg et al., 2013), the following control variables were also included: private sector provision, contributions from public sector research organizations, formal permission for redistribution and reception from other culture collections and the international orientation of the collections.

### 4.3 Data analysis method

The decision to share can reasonably be represented by binary response variables (closed question 9 of the questionnaire), but this decision varies with the source of the material. The main three sources of materials in the sample are “other culture collections”, “government research institutions” and “universities”. We therefore modelled the decision to share materials through three outcome variables. As these decision variables are correlated, we used a multivariate probit, allowing to jointly predict the decision choice when receiving materials from one of these three sources. All explanatory and control variables of the models were derived from the close-end questions of the structured questionnaire (cf. table in Annex A and the list of close-ended survey questions in Annex C). The original survey data is available online and can be retrieved through a search for the article title on the EU open access infrastructure for research data zenodo (www.zenodo.org).

## 5 Combining internalized motivations and organizational networking for the sharing of upstream research materials

### 5.1 Presentation of the results

For the analysis of materials, we chose a multivariate probit estimation to take the correlation of error terms into account (cf. Table 2). The multivariateprobit was estimated jointly for the three dependent variables. The *P*-value of the Wald test for the overall significance of the regression is very low (0.0001), indicating that the multivariate regression is highly significant. The Likelihood ratio test of rho21 = rho31 = rho32 = 0 rejects the null hypothesis at the 1% significance level: chi2(3) = 32.5639; Prob > chi2 = 0.0000. The *P*-value of the probit estimation for early data release is very low (0.0000), also indicating a highly significant regression (cf. Table 3). Conventional collinearity tests amongst the explanatory and control variables were conducted within Stata and showed no sign of collinearity amongst the variables (mean variance inflation factor (VIF) = 1.27 and 1.17 for the regression on materials and genomic data, respectively; SQRT VIF below 1.5 for all variables).

### 5.2 Discussion of the results

In the three sub-sections below, we first discuss the variables that are significant at the 1% or the 5% level for at least two of the three outcome variables in the model for sharing of materials (MSHARING ALLOW) and for the single outcome variable in the model for public availability of data (EARLY DATA RELEASE). The last sub-section then shortly discusses the remaining variables that are weakly significant in one of the two models and which have not been addressed before.

#### 5.2.1 Most significant variables of the social production model

The general outcome of the survey confirms the hypothesis that a specific set of social motivation features underlie the functioning of global knowledge commons. Indeed, in case of materials, the variables that play a role both in the social production model and the collaborative economy model (internalized motivation and non-profit orientation) are significantly correlated with sharing of materials. In the case of data, only the non-profit orientation is highly significant, and not the internalized motivations. The latter might reflect the higher degree of rents that can be obtained from data secrecy, and the lesser expected contextual benefits from data release as discussed above.

#### 5.2.2 Most significant variables related to the organizational networking, the competitive pressures and the access and benefit sharing regime

To account for the observed level of sharing of materials, the model needs to be completed however. In the case of materials, internalized motivations alone are not sufficient to account for the observed level of sharing amongst the collections. The observed levels of sharing are reached when one combines internalized motivations and organizational networking. Indeed, for all the outcome variables, the percentage of sharing behaviour in the sub-group of respondents that replied positively to “Materials prov duty” (value = 1), but which are not member of any network (value = 1 for “Networks no”), is substantially less as compared to the remaining organizations in the sample (for this sub-group the percentage decreases respectively with 11%, 23% and 23% for the outcome variables received from cc, received from gov res instit and received from univ, as compared to the remaining organizations). In addition, the combined model provides better results than alternative models that estimate the decision to share materials based on the social production or on the collaborative economy model alone. For instance, a model without the variable “Materials prov duty” but with the variable “Networks no”, or a model without the variable “Networks no” but with “Materials prov duty”, provides less significant estimations on the core explanatory variables as compared to the combined model of Table 2 (we omitted two control variables “Home contribute” and “Receive governt funded large” in this comparative exercise, in order to reach convergence of the multivariate probit estimation for all the tested models).

These results on the sharing of materials are consistent with the collaborative economy model of the sharing of excess capacity, which points to the role of organizational networks in selecting trustworthy users and providers of excess capacity. The networks that play a role in the case of the culture collections are the World Federation and the 5 regional federations that closely collaborate with the World Federation (respectively the 2 Asian federations, the European Federation, the North American Federation and the South American Federation (acronyms cf. table in Annex A).

In the case of data, as discussed above, competitive pressures amongst scientists tend to play an important role in the trade-off with the social production model, as can be witnessed from the significant positive correlation with external incentives (Publishers’ rule) and negative correlation with the consultation of the scientists (Consult scientists).

Finally, the impact of access and benefit-sharing (ABS) rules on the scientific research commons shows a clear contrast between the case of data and materials. Indeed, the existence of ABS rules does not have a highly significant impact on the sharing of materials, while ABS rules related to data are correlated to a significant decrease in public data deposit (both for “Data ABS country” and “Data ABS org”). This result can be related to the legal uncertainty surrounding the impact of ABS on data in the collections, in contrast to the case of materials where material transfer agreements for ABS have been discussed and implemented for a long time.

#### 5.2.3 Most significant control variables

The relevance of these results for the discussion of the global regulation of scientific research commons is further underlined by the analysis of the control variables.

First, increased sharing of materials is correlated with a more international orientation in the acquisition of new materials (“Collection abroad”). Moreover, the variable that controls for the presence of type strains or other reference strains in the recipient collection (“Formal third party use for some”) is significant and positive as can be expected from the previous studies. Indeed, such formal contracts are specifically important for type strains (cf. the regression results in Stromberg et al., 2013), as these reference taxonomic strains should be available for the community in compliance with the rules of the World Federation of Culture Collections. Furthermore, the variable “Receive govern funded large” (receiving over 50 samples a year from government institutions) is indicative for collections with large storage capacity (Dedeurwaerdere et al., 2012b). Deposits of samples for further downstream distribution is likely to be done in priority in such larger collections, which often have more resources to organize distribution and long-term storage of samples.

Second, the control variable “Contributiongd large” is significant and positively correlated with the early release of data, which is consistent with the importance of human resources for organizing data contribution (Tenopir et al., 2011). The same is true for the non-OECD/non-BRIC organizations (“NonOECD/BRIC”), which can be presumed to have less capacity for data contribution.

#### 5.2.4 Some remaining weakly significant variables

For materials, the variable “Home Contribute” is only significant in the case of culture collections. This might mean that in the case of culture collections, less permission is given for downstream sharing to recipients for collections that do not have a clear international service orientation. The absence of provision to private sector companies (“private sector no”) is negatively correlated to the permission for further downstream sharing for materials when these are received from culture collections. This latter feature is in line with the results of a previous survey that has shown that the private sector is a key user of public good services of the culture collections (Stromberg et al., 2013), especially of the taxonomic reference organisms (type strains) and reference organisms used for regulatory purposes. These general purpose organisms are amongst the most important category of organisms that are exchanged as shareable goods.

Finally, the variable “Gov rules materials” is only significant for materials received from other culture collections. The impact of the government policies on the permission to share is therefore not consistent amongst all kind of organizations. This might indicate the weak importance of this variable, but is also in line with the heterogeneous institutional nature of the organizations in the sample.

## 6 Options and best practices for public availability of upstream research assets

The survey results confirm the importance of the social production model for public availability of basic research assets in life science research. Three features that were highlighted in this study seem especially relevant in the overcoming of the challenges to the public availability of upstream research assets. First, seen the important role of the organizational networks in the decentralized monitoring of the quality of the research materials that are shared amongst the collections, a further strengthening of these networks is likely to be a crucial component of any cost-effective institutionalization. Second, both for materials and data, internalized motivations related to the contribution to the overall science fabric are more important than direct reciprocity or personal monetary gain. Therefore, commons are unlikely to thrive in the absence of soft law arrangements like codes of conduct and community norms that build consensus on the core values of the system. Third, scientists and managers seem to be more reluctant to publicly release data, as compared to the sharing of excess capacity in materials, in the absence of strong governmental/research funders’ incentives or regulations. Such differences between the role of external regulators in the data and materials commons could also lead to envision different pathways for further development of public research infrastructure in these two domains.

Although a more detailed institutional analysis would be required, the general features that result from this study provide some indications for best practice guidelines for a mutually supportive implementation of the Protocol on the one hand and the further institutionalization of the public research infrastructures in upstream research assets on the other. First, approximately half of the collections contribute to the global science commons by allowing further downstream sharing of research materials by qualified collections for handling microbial materials (between 42 and 62%, cf. table in Annex A). In order to comply with the tracking and monitoring obligations of the Protocol, a further refinement of this system will probably be required (Reichman et al., 2015). Some prominent initiatives have already implemented such a system, such as the European Culture Collection Organisations’ standard MTA (Cf. www.eccosite.org) and the model agreement adopted by the EU MICRO B3 Consortium for marine microbial research (von Kries et al., 2015). Second, the main motivational drivers of the research commons are related to norms and social networks, not to direct reciprocity. Therefore, the further investment in codes of conducts and guidelines that integrated ABS concerns seems a crucial component for a mutually supportive implementation. Finally, the legal uncertainty surrounding the issue of access to upstream research data appears to have a negative impact on early data release. Therefore, a further clarification of the legal issues and the development of possible legal models for public availability of upstream research data under the Protocol, with a view to reducing such uncertainty, will be required for a long-term institutionalization of the research commons.

## 7 Conclusion

This paper analyzed the barriers and opportunities for building global scientific research commons under the Nagoya Protocol, through a specific case study of the global sharing of microbial assets and associated microbial genomic data. Overall, the study confirms the hypotheses of the social production model of information and the collaborative economy model of the sharing of excess capacity in research materials. These models are an important type of non-commercial exchange of research materials and associated data, which is an important field of legislative activity under the implementation of the Nagoya Protocol. Therefore, an attention to the specific motivational and transaction costs features of these commons will be crucial in a mutually supportive institutionalization of international public research infrastructures and the implementation of the Protocol.

## Figures and Tables

**Table 1:** Comparison between the research sample and the overall population of collections.

	Population of WFCC members (626)	Research sample (191)
*Type of collection*		
Governmental	41%	41%
University	43%	40%
Semi-governmental	6%	9%
For profit collection	5%	6%
Industry collection	5%	4%

*Geographical situation*		
Asia	34%	39%
Europe	34%	31%
America	24%	27%
Oceania	6%	1%
Africa	2%	2%

**Table 2:** Results of the multivariate probit estimation on the sharing of microbial materials. For the definition of the variables, descriptive statistics and correlation matrix, cf. Annexes A–C.

Outcome variable: MSHARING ALLOW (*n* = 120)										
		Received from cc			Received from gov res instit			Received from univ		
		Signif.	Coef.	Std. err.	Signif.	Coef.	Std. err.	Signif.	Coef.	Std. err.
*Explanatory variables*										
Non-profit orientation	For profit td:paraenter	(−)	−6.6761	464	(−)**	−2.8107	1.0883	(−)**	−1.6868	0.7661
Organizational network membership	Networks no td:paraenter	(−)*	−0.8956	0.4630	(−)**	−0.8711	0.4383	(−)***	−0.9898	0.3585
Internalized motivations	Materials prov duty	(+)*	0.5923	0.3396	(+)***	1.3029	0.3492	(+)***	0.9318	0.2974
External rules/incentives for sharing	Materials ABS country	(+)	0.2395	0.3702	(+)**	0.8921	0.4328	(+)	0.0389	0.3600
	Materials ABS org	(−)	−0.0212	0.3891	(−)	−0.0025	0.4010	(−)	−0.0611	0.4195
	Gov incentives materials	(+)	0.5883	0.3723	(+)	0.0490	0.3862	(+)	0.4395	0.3617
	Gov rules materials	(−)**	−1.0696	0.4602	(−)	−0.7146	0.4515	(+)	0.0444	0.3780

*Control variables*										
Provenance of materials	Other cc	(+)	0.2456	0.5735	(−)	−0.4834	0.4356	(−)	−0.2961	0.3808
	Collection abroad	(+)**	1.7059	0.7099	(+)**	1.0249	0.5048	(+)***	1.1586	0.4048
Size/nature of contributions of materials	Home contribute	(−)**	−1.4521	0.7102	(+)	4.9234	366	(+)	1.2131	1.0633
	Private sector no	(−)***	−1.1596	0.3854	(−)	−0.3371	0.3382	(−)	−0.4697	0.3066
	Formal third party use for some	(+)**	0.7084	0.3430	(+)**	0.7741	0.3330	(+)**	0.6777	0.2986
Capacity	Receive governt funded large	(+)**	1.8068	0.7751	(+)**	2.6364	1.0988	(+)	4.5865	526

* Significant at 10% level.

** Significant at 5% level.

*** Significant at 1% level.

**Table 3:** Results of the probit estimation on the early release of genomic data. For the definition of the variables, descriptive statistics and correlation matrix, cf. Annexes A–C.

Outcome variable: EARLY DATA RELEASE (*n* = 112)				
		Signif.	Coef.	Std. err.
*Explanatory variables*				
Non-profit orientation	For profit	(−)**	−1.3077	0.6267
Internalized motivations	Data prov duty	(+)*	0.6242	0.3343
External rules/incentives for sharing	Data ABS country	(−)***	−0.9723	0.3440
	Data ABS org	(−)***	−1.2133	0.3226
	Publishers’ rules	(+)**	0.5718	0.2887
	Perc employers role	(+)	0.5503	0.3793
	Gov rules data	(+)	0.3843	0.3755
	Consult nat public authorities	(+)	0.4418	0.2854
	Consult scientists	(−)**	−1.0179	0.3994

*Control variable*s				
Provenance of materials	Collect abroad	(+)	0.3017	0.3681
Size/nature of data contributions	Contributiongd large	(+)***	1.3858	0.2792
Capacity	NonOECD/BRIC	(−)**	−0.6115	0.3008

* Significant at 10% level.

** Significant at 5% level.

*** Significant at 1% level.

## Appendix A Supplementary data

Annex A Definition of the variables and descriptive statistics.

Annex B Correlation matrix amongst the independent variables.

Annex C Questions of the close-ended questionnaire.

## Footnotes

Appendix A Supplementary data associated with this article can be found, in the online version, at http://dx.doi.org/10.1016/j.envsci.2015.08.006.
