# Supplementary material for: Global scientific research commons under the Nagoya Protocol: Towards a collaborative economy model for the sharing of basic research assets
Source: Environ Sci Policy. 2016 Jan;55:1–10. doi: 10.1016/j.envsci.2015.08.006 (PMC5268345; doi:10.1016/j.envsci.2015.08.006)
Supplement: Annex A — Definition of the variables and descriptive statistics. [file mmc1.docx]

**Annex A. Definition of the variables and descriptive statistics**

Probit estimation for Materials (n = 120)

| **OUTCOME** | | mean | Std. Dev. | Min-max | Survey question |
| --- | --- | --- | --- | --- | --- |
| Msharing allow: received from culture coll | =1 if redistribution of microbial cultures is allowed when these are received from other culture collections  = 0 not allowed/uncertain | 0,42 | 0,5 | 0-1 | 9 |
| Msharing allow: received from gov res instit | =1 if redistribution of microbial cultures is allowed when these are received from government funded research institutes  = 0 not allowed/uncertain | 0,53 | 0,5 | 0-1 | 9 |
| Msharing allow: received from univ | =1 if redistribution of microbial cultures is allowed when these are received from universities  = 0 not allowed/uncertain | 0,62 | 0,49 | 0-1 | 9 |
| **EXPLANATORY** | |  |  |  |  |
| For profit | =1 the organization is a for profit research institute, a for profit culture collection or a private company  =0 the organization is a government funded research institute, a government funded culture collection, a research institute at a university or culture collection at a university | 0,05 | 0,22 | 0-1 | 2 |
| Networks no | =1 does not belong to one of the networks of world or regional culture collections federations (WFCC, ECCO, FELACC, USCCN, ACM, ANRRC, ABRCN)**  =0 belongs to one of these networks | 0,21 | 0,41 | 0-1 | 26 |
| Materials prov duty | =1 duty ranked first amongst 4 factors that play a role in the decision to provide materials  =0 another factor ranked first: research partnerships, commercialization of new products, provision of materials to me | 0,65 | 0,48 | 0-1 | 17 |
| Materials ABS country | = 1 if general ABS rules in my country apply to me  = 0 if this option is not selected | 0,29 | 0,46 | 0-1 | 22 |
| Materials ABS org | = 1 if ABS policy of “my” organization applies to me  = 0 if this option is not selected | 0,25 | 0,43 | 0-1 | 22 |
| Gov incentives materials | =1 receives « occasionally » or « regularly » benefits from incentives or direct payments from government bodies, when providing materials to others  = 0 never receives such benefits, when providing materials to others | 0,22 | 0,42 | 0-1 | 18 |
| Gov rules materials | = 1 the Rules imposed by the Government determine the decision on the conditions of transfer of material  =0 case by case decision by individuals or centralized decision by a manager determine the decision on the conditions of transfer of material | 0,19 | 0,4 | 0-1 | 23 |
| **CONTROL** | |  |  |  |  |
| Other cc | = 1 the organization received samples from other culture collections during the 10 most recent years of activity  =0 the organization does not receive samples from other culture collections | 0,87 | 0,34 | 0-1 | 4 |
| Collection abroad | =1 if approximately 20% or more of the samples are received / collected from abroad  = 0 if none of the samples are received / collected from abroad | 0,83 | 0,37 | 0-1 | 6 |
| Receive govern funded large | =1 if more than 50 samples are received from government funded research institutes  = 0 if 50 samples or less are received from government funded research institutes | 0,11 | 0,31 | 0-1 | 4 |
| Home contribute | =1 the organization provides samples within the home country (approximately 20% or more of all samples provided)  =0 the organization does not provide samples within the home country | 0,92 | 0,28 | 0-1 | 13 |
| Formal third party use for some | =1 if authorisation for redistribution to third parties is given to at least one category of recipient of the distributed materials  = if no category of recipient receives such an authorisation | 0,54 | 0,5 | 0-1 | 15 |
| Private sector no | = 1 you do not provide materials to private sector company  =0 you provide materials to private sector company | 0,35 | 0,48 | 0-1 | 12 |

Probit estimation for genomic data (n = 112)

| **OUTCOME** | | mean | Std.Dev. | MinMax | Survey question |
| --- | --- | --- | --- | --- | --- |
| Early data release | =1 if data is submitted to public online genomic databases immediately after the generation of the data, at the time of deposit of materials or at the time of publication of a paper based on that data  =0 if data is submitted after the publication of all papers based on that data or never | 0,59 | 0,5 | 0-1 | 20 |
| **EXPLANATORY** | |  |  |  |  |
| For profit | =1 the organization is for profit research institute or for profit culture collection or private company  =0 the organization is government funded research institute, government funded culture collection, research institute at a university, culture collection at a university | 0,05 | 0,23 | 0-1 | 2 |
| Data prov duty | =1 if duty ranked 1st or 2^nd^ amongst 4 factors that motivate data provision to public online databases by the respondents  =0 if duty ranked 3^rd^ or 4^th^ (other options : reputation in the scientific community, mandatory for publications, mandated by funding agency) | 0,58 | 0,49 | 0-1 | 21 |
| dataabscountry | =1 access and benefit sharing policies concerning access and use of data decided by my country apply to me  = 0 access and benefit sharing policies decided by my organization apply to me or I don’t know | 0,22 | 0,42 | 0-1 | 22 |
| dataabsorg | =1 access and benefit sharing policies concerning access and use of data decided by my organization apply to me  = 0 access and benefit sharing policies decided by my country apply to me or I don’t know | 0,29 | 0,45 | 0-1 | 22 |
| Publishers’ rules | = 1 if « mandatory for publications » ranked 1st or 2^nd^ amongst 4 factors that motivate data provision to public online databases  =0 if « mandatory for publications » ranked 3^rd^ or 4^th^ (other options : reputation in the scientific community, duty, mandated by funding agency) | 0,82 | 0,38 | 0-1 | 21 |
| Perc employers role | =1 if promotion of data sharing by employers is ranked 1^st^ or 2^nd^ amongst 4 factors that are important for creating more willingness for data provision  = 0 if ranked 3^rd^ or 4^th^  (other options: meetings with other researchers/collections; legal certainty; transparency on transactions; promotion by funding agencies) | 0,18 | 0,38 | 0-1 | 25 |
| Consult scientists | = 1 if scientists within your organization are consulted (occasionally or always) while making decisions on the conditions of transfer of data  =0 if they are never consulted for such decisions | 0,91 | 0,29 | 0-1 | 24 |
| Gov. rules data | =1 if government determines the decisions on the conditions of transfer of data in my organization  = 0 if individuals/managers determine such decisions | 0,13 | 0,34 | 0-1 | 23 |
| Consult nat public authorities | =1 if national public authorities are consulted (occasionally or always)while making decisions on the conditions of transfer of data  =0 if they are never consulted | 0,62 | 0,49 | 0-1 | 24 |
| **CONTROL** | |  |  |  |  |
| Collect abroad | = 1 if 20% or more are collected abroad  =0 none are collected abroad | 0,81 | 0,39 | 0-1 | 6 |
| Contributiongd large | =1 if the frequency of contribution of data to genomic databases is once in a month or several times each month  =0 if the frequency of contribution of data to genomic databases is never/once in a year or twice in a year | 0,47 | 0,5 | 0-1 | 19 |
| NonOECD/BRIC | = 1 if the country is not an OECD country or a BRIC country (Brasil, India, China)  = 0 if the country is an OECD country or a BRIC country (Brasil, India, China) | 0,27 | 0,44 | 0-1 | 3 |

q*: question number of the survey

(WFCC, ECCO, FELACC, USCCN, ACM, ANRRC, ABRCN)**

WFCC: World Federation for Culture Collections

ECCO: European Culture Collection Organisation

FELACC Federación Latinoamericana de Colecciones de Cultivos

USCCN : United States Culture Collection Network

ACM: Asian Consortium for the Conservation and Sustainable Use of Microbial Resources (ACM)​

ANRRC: Asian Network of Research Resource Centers

ABRCN: Asian Biological Resource Center Network
