# Supplementary material for: Global scientific research commons under the Nagoya Protocol: Towards a collaborative economy model for the sharing of basic research assets
Source: Environ Sci Policy. 2016 Jan;55:1–10. doi: 10.1016/j.envsci.2015.08.006 (PMC5268345; doi:10.1016/j.envsci.2015.08.006)
Supplement: Annex B — Correlation matrix amongst the independent variables. [file mmc2.docx]

**Annex B. Correlation matrix amongst the independent variables**

Correlation matrix for the multivariate probit estimation on the sharing of microbial materials

Correlation matrix for the probit estimation on the early release of genomic data
