# Supplementary material for: Global scientific research commons under the Nagoya Protocol: Towards a collaborative economy model for the sharing of basic research assets
Source: Environ Sci Policy. 2016 Jan;55:1–10. doi: 10.1016/j.envsci.2015.08.006 (PMC5268345; doi:10.1016/j.envsci.2015.08.006)
Supplement: Annex C — Questions of the close-ended questionnaire. [file mmc3.docx]

**Personal and institutional profile**

Please note that all the information you enter here will be treated with full confidentiality and will be used solely for statistical purposes.

**1. Which of the following best describes your profession? (You may select more than one option)**

Culture Collection Manager (A person whose duties are mainly related to managerial activities at the collection) Academician (A person whose duties are mainly related to teaching/ training activities at an educational institution) Researcher (A person whose duties are mainly related to research activities)

Other (please specify)

**2. Which of the following best describes your organisation? (You may select more than**

**one option)**

Government funded research institute Government funded culture collection For profit research institute

For profit culture collection Research institute at a university Culture collection at a university Private company

Other (please specify)

**3. In which country is your organisation located:**

**Receiving materials**

**4. How many samples of MICROBIAL CULTURES do you receive PER YEAR (on**

**average: based on your 10 most recent years of activity or just your most recent years of activity if less) from the following sources:**

None 1-5/ year

6-10/

year

11-15/

year

16-20/

year

21-25/

year

26-30/

year

31-35/

year

36-40/

year

41-45/

year

46-50/

year

> 50/

year

Directly collected in nature Other culture collections Universities

Government funded research

institutes

Private sector companies Hospitals Other

**5. How many samples of DNA materials that are not culturable micro-organisms do you**

**receive PER YEAR (on average: based on your 10 most recent years of activity or just your most recent years of activity if less) from the following sources:**

None 1-5/ year

6-10/

year

11-15/

year

16-20/

year

21-25/

year

26-30/

year

31-35/

year

36-40/

year

41-45/

year

46-50/

year

> 50/

year

Directly collected in nature Other culture collections Universities

Government funded research

institutes

Private sector companies Hospitals Other

**6. Could you please indicate the relative percentages (approximately!) of the materials**

**that you receive or collect from within the home country of your organisation, as compared to those you receive or collect from abroad (Total: 100%)**

None 20% 40% 60% 80% 100%

from the home country of your organisation

from abroad

**7. If you are also receiving materials from abroad, could you please indicate the relative percentages (approximately!) of the following geographical regions in the total materials you receive or collect from abroad (Total: 100%):**

None 10% 20% 30% 40% 50% 60% 70% 80% 90% 100%

Africa

America

Asia

Europe

Oceania (Australia, New Zealand and Papua New Guinea)

Antartica

**8. What percentage (approximately) of the following materials you receive or collect are**

**generally stored for long term conservation (more than 10 years):**

None 1-20% 21-40% 41-60% 61-80% 81-100%

Microbial cultures

DNA materials that are not culturable micro-organisms

**9. Are you generally allowed to redistribute the MICROBIAL CULTURES, if they were**

**RECEIVED from the following:**

No Yes, I can redistribute them I don't know/ Not applicable

Other culture collections

Government funded research institutes

Universities

Private Sector companies

Hospitals

Closely related collaborators

**10. Are you generally allowed to redistribute the DNA MATERIALS that are not**

**culturable micro-organisms, if they were RECEIVED from the following:**

Other culture collections

No Yes, I can redistribute them I don't know/ Not applicable

Government funded research institutes

Universities

Private Sector companies

Hospitals

Closely related collaborators

**Providing materials**

**11. How many samples of the following materials do you provide PER YEAR (on**

**average: based on your 10 most recent years of activity or just your most recent years of activity if less) to persons outside your organisation (for example, other researchers, collection managers, academicians, etc.):**

Microbial cultures

None 1-5/ year

6-10/

year

11-15/

year

16-20/

year

21-25/

year

26-30/

year

31-35/

year

36-40/

year

41-45/

year

46-50/

year

> 50/

year

DNA materials that are not culturable micro-organisms

**12. How many samples of materials do you provide PER YEAR to the following (on**

**average: based on your 10 most recent years of activity or just your most recent years of activity if less):**

None 1-5/ year

6-10/

year

11-15/

year

16-20/

year

21-25/

year

26-30/

year

31-35/

year

36-40/

year

41-45/

year

46-50/

year

> 50/

year

Other culture collections

Universities

Government funded research institutes

Private sector companies

Hospitals

Other

**13. Could you please indicate the relative percentages (approximately!) of providing**

**materials within the home country of your organisation, as compared to providing materials abroad (Total: 100%)**

None 20% 40% 60% 80% 100%

Provision within the home country of your organisation

Provision abroad

**14. If you are also providing materials abroad, could you please indicate the relative**

**percentages (approximately!) of the following geographical regions in the total materials provided abroad (Total: 100%):**

None 10% 20% 30% 40% 50% 60% 70% 80% 90% 100%

Africa

America

Asia

Europe

Oceania (Australia, New Zealand and Papua New Guinea)

Antartica

**15. If you are PROVIDING MICROBIAL CULTURES to the following categories of organisations, are the recipients generally allowed to REDISTRIBUTE the received materials to third parties:**

No Yes, they can redistribute them I don't know/ Not Applicable

Other culture collections

Government funded research institutes

Universities

Private Sector companies

Hospitals

Closely related collaborators

Others

**16. If you are PROVIDING DNA materials that are not microbial cultures to the following**

**categories of organisations, are the recipients generally allowed to REDISTRIBUTE the received materials to third parties:**

Other culture collections

No Yes, they can redistribute them I don't know/ Not Applicable

Government funded research institutes

Universities

Private Sector companies

Hospitals

Closely related collaborators

Others

**About motivations and benefits**

**17. Could you please indicate the relative importance of the following factors (Rank 1**

**indicates the highest importance, while Rank 4 indicates the lowest importance) in your decision to provide materials to others?**

**(Please note that the system automatically re-arranges the list of factors in the order of**

**your ranking, whenever you assign/ change the rank for any of the factors listed)**

It is my duty, as it is part of science N/A

It provides possibilities to build new research partnerships N/A

It provides the possibilities to commercialise new products and earn royalties on them N/A

Those who are receiving materials from me may provide materials to me in the future N/A

**18. How frequently do you receive the following benefits, when you provide materials to**

**others:**

Proper attribution to you (or your organisation) during any use of the materials

Never Occasionally Regularly

Fiscal incentives or direct payments from governmental bodies that incentivize material sharing

Fiscal payments in return for sharing the materials

Royalties and/ or revenues from the sale/commercialization of materials

New research contracts

Positive words from colleagues/ other researchers

**Contribution of data associated with materials**

Note: By 'data' we mean genomic, observatory or experimental data associated with microbial cultures and/or DNA

materials that are not culturable micro-organisms

**19. Which of the following best describes the frequency of your contribution of data, to the following types of public online databases:**

Culture collection databases

Never Once in a year Twice in a year Once in a month

Several times each month

Genomic databases

Other online databases

**20. In general, at what point do you submit data to the following types of public online**

**databases:**

Immediately after

At the time of deposit

At the time of

After the publication

generation of the data

of materials

publication of a paper of all papers based on

Never

Culture collection databases

based on that data

that data

Genomic databases

Other online databases

**21. Could you please indicate the relative importance of the following factors (Rank 1**

**indicates the highest importance, while Rank 4 indicates the lowest importance) in your decision to provide DATA to public online databases**

**(Please note that the system automatically re-arranges the list of factors in the order of**

**your ranking, whenever you assign/ change the rank for any of the factors listed)**

I think it is my duty as a scientist to provide DATA to public online databases N/A

It helps in increasing my reputation within the scientific community N/A

It is mandatory for publications N/A

It is mandated by my funding agency/ employer N/A

**About laws and regulations in the area of material and data sharing**

**22. Are there any official Access and Benefit Sharing (ABS) policies that applies to you**

**on the following aspects (You may select more than one option):**

ABS policy of my organisation General ABS rules in my country I don't know

Exchange of microbial cultures

Exchange of DNA materials that are not culturable microorganisms

Access and use of data and information associated to the materials held at your organisation

**23. Within your organisation, who/ what determines the decisions on the following**

**aspects:**

Conditions for transfer of materials

Case by case decision by individuals

Centralized decision by a manager of your organisation

Rules imposed by the government

Conditions for transfer of data

**24. According to you, how frequently are the following categories of people consulted**

**while making the decisions of your organisation, on the conditions for transfer of materials and/or data**

Scientists from within your organization

Never consulted Occasionally consulted Always consulted

Scientists from outside your organization

National public authorities (For example, government ministeries)

Representatives of private sector enterprises

**Factors that can promote sharing**

**25. According to you, how important are the following (Rank 1 indicates the highest**

**importance and Rank 5 indicates the lowest importance) for creating more willingness among researchers to provide materials and data to other researchers and collections: (Please note that the system automatically re-arranges the list of factors in the order of your ranking, whenever you assign/ change the rank for any of the factors listed)**

Meetings with other researchers/collections at global and regional conferences

Legal certainty on transactions with regard to materials and data

Transparency on transactions with regard to materials and data

Promotion of data/ material sharing by employers

Promotion of data/ material sharing by funding agencies

**26. Does the collection you work in belong to one or several of the following networks?**

WFCC ECCO FELACC USCNN ACM ANRRC ABRCN

none of these

no answer

**27. In what region of the world your collection is situated?**

Africa America Asia Europe

Oceania (Australia, New Zealand and Papua New Guinea) Antartica
